# Regulation of IgA Production by Intestinal Dendritic Cells and Related Cells

**DOI:** 10.3389/fimmu.2019.01891

**Published:** 2019-08-13

**Authors:** Hiroyuki Tezuka, Toshiaki Ohteki

**Affiliations:** ^1^Department of Cellular Function Analysis, Research Promotion and Support Headquarters, Fujita Health University, Aichi, Japan; ^2^Department of Biodefense Research, Medical Research Institute, Tokyo Medical and Dental University (TMDU), Tokyo, Japan

**Keywords:** dendritic cells, IgA, intestine, commensal bacteria, conditioning

## Abstract

The intestinal mucosa is a physiological barrier for most microbes, including both commensal bacteria and invading pathogens. Under homeostatic conditions, immunoglobulin A (IgA) is the major immunoglobulin isotype in the intestinal mucosa. Microbes stimulate the production of IgA, which controls bacterial translocation and neutralizes bacterial toxins at the intestinal mucosal surface. In the intestinal mucosa, dendritic cells (DCs), specialized antigen-presenting cells, regulate both T-cell-dependent (TD) and -independent (TI) immune responses. The intestinal DCs are a heterogeneous population that includes unique subsets that induce IgA synthesis in B cells. The characteristics of intestinal DCs are strongly influenced by the microenvironment, including the presence of commensal bacterial metabolites and epithelial cell-derived soluble factors. In this review, we summarize the ontogeny, classification, and function of intestinal DCs and how the intestinal microenvironment conditions DCs and their precursors to become the mucosal phenotype, in particular to regulate IgA production, after they arrive at the intestine. Understanding the mechanism of IgA synthesis could provide insights for designing effective mucosal vaccines.

## Introduction

The intestine is the largest mucosal tissue in the human body and is composed of the small intestine, caecum, and large intestine. Dietary components are absorbed and digested at the intestinal mucosa, which has a surface area of ~400 m^2^ (1). This tissue contains the largest number of immune cells and 10^14^ commensal bacteria, consisting of 500–1,000 different species (2), which cooperatively maintain intestinal homeostasis.

Since the intestinal mucosa covered with epithelial monolayer limits the invasion of commensal bacteria or pathogens, it serves as a first line of defense for the body (1). The homeostatic mucosal defense consists of at least two distinct barriers: epithelial and immunological barriers. The epithelial barrier prevents the systemic invasion of microbes by tight junctions, mucus coating, and antimicrobial peptide secretion by intestinal epithelial cells (iECs). On the other hand, the immunological barrier is largely accomplished by immunoglobulin A (IgA) antibodies, which prevent microbes from binding to the iECs, suppress the microbes' growth and virulence, and neutralize their toxins. Paradoxically, these barriers are fully developed and maintained by the continuous stimulation with commensal bacteria.

In humans and mice, ~80% of the total plasma cells in the body are located in the intestinal mucosa, where they secrete dimeric IgA under steady-state conditions (40–60 mg/kg/day or 3–5 g/day in human) (3, 4). Interestingly, circulating IgA is mainly monomeric in humans, but largely oligomeric, composed of 2–4 monomers, in mice. In addition, unlike mouse IgA, human IgA is composed of two subclasses, IgA1 and IgA2, and the latter is predominant in the mucosa (3). Homeostatic IgA (which also functions as a natural antibody) is induced in the intestinal mucosa by continuous stimulation with commensal bacteria, and is also detectable in circulation. Indeed, little IgA is detected in the intestinal secretions and sera of germ-free and neonatal mice and its production is restored soon after the colonization of commensal bacteria (2), indicating that commensal bacteria induce the development of the mucosal immune system. The homeostatic IgA is basically poly-reactive with low affinity, which can bind to common antigens on the microbes, including lipopolysaccharides, capsular polysaccharides, and flagellin; notably, some commensal bacteria (20–50%) are coated with the IgA (5, 6). Mice that lack secretory IgA exhibits biased composition of commensal bacteria, known as dysbiosis, suggesting that IgA regulates the absolute number and diversity of the commensal bacteria through their growth inhibition and elimination (7–9). In contrast, some IgA-coated bacteria can form colonies within the mucus layer to secure a niche segregated from competing species (10). Interestingly, commensal bacteria coated with high levels of IgA are more pathogenic than low IgA- and non-coated bacteria, in an induced colitis model (8).

Dendritic cells (DCs), which are widely distributed throughout organs and tissues, are specialized antigen-presenting cells with dual roles in inducing tolerance to self-antigens and inducing immunity to non-self antigens (11). The DCs in the intestine are preferentially localized beneath the epithelial monolayer, where they detect luminal antigens, including commensal bacteria, dietary antigens, and damaged iEC-associated components, to survey luminal environments. As intestinal DCs continually sample luminal antigens in the steady-state, they appear to be tolerogenic. Indeed, intestinal DCs that have engulfed orally administered antigens or apoptotic epithelial components migrate into the draining mesenteric lymph nodes (MLNs), where they suppress immune responses against these antigens through the induction of CD4^+^Foxp3^+^ regulatory T cells (Tregs) (12). In addition, intestinal DCs carrying live commensal bacteria induce IgA class switching in B cells after they arrive at the MLNs (13). Thus, DCs are integrally involved in maintaining intestinal homeostasis by transporting luminal antigens to the draining lymph nodes. The unique functions of DCs are thought to be acquired under the influence of intestinal microenvironments.

Here we review and discuss current understanding of the ontogeny and conditioning of and regulation of IgA synthesis by DCs in the intestinal microenvironments, largely based on the knowledge obtained from mouse models.

## Galt and Related Tissues

The gut-associated lymphoid tissues (GALT) include Peyer's patches (PPs) and isolated lymphoid follicles (ILFs) (14). Both are covered with an epithelial monolayer called the follicle-associated epithelium (FAE), which contains microfold cells (M cells) that are specialized for antigen uptake, but are not connected to the afferent lymphatics (14). In the PPs, B cell follicles are covered by subepithelial dome (SED) that lies beneath the FAE, and are surrounded by T cell zones (interfollicular regions; IFR). The SED contains numerous DCs, which engulf luminal antigens such as live microbes and undigested dietary antigens. In comparison with the PPs, ILFs have no distinct T cell zone and B cell follicles are relatively smaller and less mature.

Once having expressed gut-homing receptors, class-switched B cells and effector T cells leave the PPs or ILFs and migrate into the draining MLNs via the lymphatics. In this process, all-*trans*-retinoic acid (RA) produced by DCs and stromal cells (SCs) in the MLNs induces gut-homing receptor CCR9 and integrin α4β7 on the lymphocytes (15, 16), so called “imprinting.” After circulating through the thoracic duct and blood, they migrate back to the intestinal lamina propria (LP), a connective tissue. During the homing process, CCR9 and the integrin α4β7 bind to CCL25 produced by iECs and mucosal addressin cell adhesion molecule 1 (MAdCAM-1) on endothelial cells of the intestine, respectively (17, 18), leading to their successful homing to the LP. In addition, DCs in the PPs, ILFs, and LP migrate to the MLNs in a CCR7-dependent manner, where they present luminal antigens to naïve T cells (12, 13). Unlike lymphocytes, DCs do not exit from the MLN to the efferent lymphatics at least under steady-state conditions (13), indicating that the MLNs function as a “firewall” to prevent the penetration of luminal antigen-laden DCs into the periphery.

## Classification, Distribution, and Function of Intestinal Dendritic Cells

DCs consist of two subsets: the monocyte-derived, colony-stimulating factor 1/2 (CSF-1/2)-dependent DCs and the FMS-like tyrosine kinase ligand (Flt3L)-dependent DCs (19); the latter group can be classified further into classical DCs (cDCs) and plasmacytoid DCs (pDCs) (Table 1). It is often difficult to distinguish monocyte-derived DCs from macrophages based on their cell surface markers and functions (19). LP cDCs can be subdivided into at least three distinct subpopulations based on the expression of CD103 (also known as integrin αE), CD11b, XCR1, and SIRPα (also known as CD172a) (44), i.e., CD103^+^CD11b^−^XCR1^+^cDC1 (hereafter, XCR1^+^ cDC1), CD103^+^CD11b^+^SIRPα^+^cDC2 (hereafter, CD103^+^ cDC2), and CD103^−^CD11b^+^SIRPα^+^cDC2 (hereafter, CD103^−^ cDC2), all of which depend on the transcription factor Zbtb46 for their development (45, 46) (Table 1). In this regard, TFs required for these cDC subsets are as follows; BATF3, Id2, or IRF8 for XCR1^+^ cDC1 (20–22), IRF4, Notch2, or KLF4 for CD103^+^ cDC2 (25–27), Zeb2 for CD103^−^ cDC2 (30) (Table 1). Likewise, based on the expression of CD11b, CD8α, XCR1, and SIRPα, PP cDCs are subdivided into several populations (47, 48). In addition, the development of pDCs in the GALT is dependent on E2-2 and, to a lesser extent, on Zeb2 (30, 32) (Table 1).

**Table 1:** DCs and their related populations in the intestine.

**Subsets (location)**	**Progenitors in blood**	**Transcription factors**	**Cytokines**	**Functions**	**Selected references**
CD103^+^CD11b^−^ XCR1^+^cDC1 (LP)	Pre-cDC	Zbtb46 Batf3 Irf8 Id2	Flt3L	CD8^+^ T cell generation CD4^+^CD8αα^+^ IEL generation Th1 generation Treg generation Cross-presentation CCR9/α4β7 expression	(12, 20–24)
CD103^+^CD11b^+^ SIRPα^+^ cDC2 (LP)	Pre-cDC	Zbtb46 Irf4 Klf4 Notch2	Flt3L	Th17 generation Th2 generation IgA^+^ B cell generation Treg generation	(25–29)
CD103^−^CD11b^+^ SIRPα^+^ cDC2 (LP)	Pre-cDC	Zbtb46 Zeb2	Flt3L	Th17 generation	(30, 31)
CD11C^int^B220^+^ Singlec H^+^ pDC (LP, PP, MLN)	pDC	E2-2	Flt3L	Treg generation IgA^+^ B cell generation	(30, 32, 33)
Monocyte-derived CX3CR1^+^ DC (LP, PP)	Ly6C^+^ monocyte	Runx3 Irf4 Pu.1	CSF1 CSF2	Th1 generation	(34–41)
Monocyte-derived Tip-DC (LP, PP)	Ly6C^+^ monocyte	(^*^)	CSF1 CSF2	IgA^+^ B cell generation	(42, 43)

*Only intestinal DC subsets whose origin, transcription factors, cytokines necessary for differentiation and functions are clear are listed*.

* *Transcription factors required for Tip-DCs development remain unclear. LP, lamina propria; MLN, mesenteric lymph nodes; PP, Peyer's patches*.

In the GALT, cDCs and macrophages express similar surface markers (44). For example, macrophages activated in the intestine are CD11c^hi^MHC class II^hi^, which closely resemble cDCs, and CD11c^+^CX_3_CR1^hi^ cells in the LP are now regarded as macrophages rather than DCs based on their functions, ontogeny, and TF requirements. In addition, CD103^−^CD11b^+^ LP cells, originally referred to as cDCs, are turned to be composed of a large number of monocyte-derived macrophages and a small number of CD103^−^ cDC2 (31, 49, 50). In this regard, intestinal macrophages can be characterized by their expression of CD64, Mer tyrosine kinase, and CD169 in addition to a classical marker F4/80 (44). Indeed, most of the CD103^−^CD11b^+^ LP cells express these markers (31). The formal distinction of intestinal DCs and macrophages need their anatomical distribution and biological functions, in addition to their surface marker expression, as discussed below.

Among the LP DC subsets, CD103^+^ DCs, which possibly include XCR1^+^ cDC1 and CD103^+^ cDC2, are preferentially localized in the center of villus, whereas CD103^−^ DC2 reside around them (51). XCR1^+^ cDC1 are required for the generation and maintenance of intestinal intraepithelial T cells. They also cross-present iEC-derived antigens to CD8^+^ T cells and promote the differentiation of Th1 cells and Tregs in the GALT (23, 24). On the other hand, CD103^+^ cDC2 induce Th17 cells and IgA^+^ B cells under steady-state conditions (26, 28) and protective Th2 immunity to the parasitic worms, *Nippostrongylus brasiliensis* and *Schistosoma mansoni* (27, 29). Although CD103^−^ cDC2 cells are also able to induce Th17 cells at least *in vitro* (31), it remains unclear whether they contribute to intestinal Th17 cell homeostasis (Table 1). cDC2 and pDCs localized in the PPs induce IgA synthesis in a commensal bacteria-dependent manner (13, 33, 52). PP pDCs migrate into the intestinal LP in a CCR9-dependent manner (53) and maintain Tregs, leading to the induction of oral tolerance (54, 55). Interestingly, intestinal pDCs do not produce large amounts of type 1 IFNs (33, 56, 57). The role of DCs and their related cells in intestinal IgA synthesis is described later in the section “ROLE OF DENDRITIC CELLS IN INTESTINAL IGA PRODUCTION.”

### Antigen Sampling by and Trafficking of DCs

In the PPs, CCR6^+^ cDCs in the SED move into the FAE via the CCR6-CCL20 interaction to sample luminal microbes, e.g., *Salmonella typhimurium*, that invades across the FAE and then migrate into the IFR, where they activate *S. typhimurium*-specific T cells (58). Monocyte-derived CX_3_CR1^+^ cells that are defined by TFs including Rnux3/p33, IRF4, and Pu.1 are in close contact with the FAE and express lysozyme, a representative antimicrobial enzyme (34–38) (Table 1). The lysozyme^+^ CX_3_CR1^+^ cells extend dendrites into the lumen through the transcellular pores of M cells to capture and kill *S. typhimurium* (39, 59). Interestingly, some PP cDCs carrying luminal antigens migrate into the MLNs in a CCR7-dependent manner (13).

In the LP, CX_3_CR1^+^ macrophages are preferentially localized beneath the epithelial layer via the interaction with iEC-derived CX_3_CL1 (also known as fractalkine). They can directly sample luminal antigen by extending dendrites through their expression of tight junction-related proteins (60, 61) and can also sample luminal microbes transported through M cells in the villous epithelium (62). Although phagocytic activity of CX_3_CR1^+^ macrophages is much greater than CD103^+^ DCs, antigen-presenting capacity is in an opposite way (51).

These findings lead to the question how CD103^+^ DCs recognize luminal antigens. Some activated CD103^+^ DCs, which express tight junction-associated proteins, migrate into beneath the epithelial layer, where they sample luminal soluble antigens by extending their dendrites into the lumen, or engulf the antigens delivered in the LP through goblet cell transcytosis (63, 64). Intriguingly, CD103^+^ cDC2 can get indirectly luminal antigens through a membrane transfer system called trogocytosis. In brief, CD103^+^ cDC2 receive soluble antigens with some membrane from CX_3_CR1^+^ macrophages through gap-junctions formed between these cells, thereby inducting oral tolerance (65). This cooperative process may compensate for the poor phagocytic activity of CD103^+^ DCs.

After acquiring luminal dietary antigens, CD103^+^ LP DCs migrate in a CCR7-dependent manner into the MLNs via the afferent lymphatics, then present the antigens to naïve T cells (51, 66). Unlike the CD103^+^ DCs, CX_3_CR1^+^ macrophages in the LP do not migrate into the MLNs in steady-states. However, under inflammatory conditions, the CX_3_CR1^+^ LP macrophages and related cells appear to migrate into the MLNs (40, 67). During dysbiotic colitis, Ly6C^+^ inflammatory monocytes enter into the LP of inflamed colon, where they give rise to CX_3_CR1^int^CCR7^+^ macrophages that have a capacity to migrate into the MLNs (40). In addition, CX_3_CR1^+^ LP cells carrying *Salmonella* can migrate in a CCR7-dependrnt manner into the MLNs in antibiotic-treated dysbiotic mice (67), suggesting that healthy microbiota may restrict CX_3_CR1^+^ cell migration.

## Conditioning of Dendritic Cells in the Intestine

Mucosal DCs functionally differ from non-mucosal DCs, and their functional properties are likely to be influenced by the unique microenvironment at each mucosal site. Intestinal DCs are no exception and conditioned by commensal bacterial and dietary antigens directly or indirectly through iECs under steady-state conditions.

### Commensal Bacterial Conditioning of DCs

Commensal bacterial products directly condition DCs in the GALT. The human commensal bacteria *Bacteroides fragilis*-derived polysaccharide A (PSA) induces inducible nitric oxide synthase (iNOS) in monocyte-derived DCs through Toll-like receptor 2 (TLR2) signaling (68) (Table 2). In colitis models, PSA-conditioned DCs prevent the colonic inflammation by generating IL-10-producing Tregs in the MLNs (70, 77, 78). In addition, the iNOS^+^ DCs likely contribute to IgA synthesis as described in the following section.

**Table 2:** The effect of intestinal microenvironmental conditioning factors on DCs and their related populations.

**Conditioning factors**	**Target cells**	**Receptors**	**Conditioned cell-derived factors**	**Induction of effector cells**	**References**
**LIVE BACTERIA**					
*Enterobactor* spp.	PP cDC	(Phagocytosis)	Not determined	lgA^+^ B cells	(13)
*Alcaligenes* spp.	PP cDC	(Phagocytosis)	BAFF, IL-6, TGF-β	lgA^+^ B cells	(69)
**BACTERIAL PRODUCTS**					
Polysaccharide A	Mo-DC	TLR2	iNOS	Th1	(68)
Polysaccharide A	BM pDC	TLR2	MHCII, ICOSL, CD86	Treg	(70)
Butyrate	Mo-DC	GPR109A	Retinoic acid	Treg, Tr1	(71)
Acetate	Mo-DC	GPR43	Retinoic acid	lgA^+^ B cells	(72)
Lactate*I*Pyruvate	LP MP	GPR31	Not determined	(Dendrite protrusion)	(73)
**iEC-DERIVED FACTORS**					
Wnt	LP cDC	Frizzeld	Retinoic acid	Treg	(74)
	LP MP		IL-10, TGF-β		
Mucus	LP cDC	Galectin-3-Dectin-1-FcγRIIB -complex	Retinoic acid IL-10, TGF-β	Treg	(75)
Serum amyloid A	LP cDC	CD36	IL-6, IL-23	Th17	(76)
**SC-DERIVED FACTORS**					
IFN-α/IFN-β	PP/MLN	IFNAR1/IFNAR2	BAFF, APRIL	lgA^+^ B cells	(33)
	pDC				

*BM, bone marrow; GPR, G-protein-coupled receptor; iEC, intestinal epithelial cells; LP, lamina propria; MLN, mesenteric lymph node; Mo-DC; monocyte-derived DC; MP, macrophages; PP, Peyer's patch; SC, stromal cells; TLR, Toll-like receptor*.

Commensal bacteria-derived short-chain fatty acids (SCFAs), i.e., acetate, butyrate, and propionate, condition DCs. Butyrate binds to G-protein-coupled receptor 109a (GPR109a) on DCs to generate RA-producing DCs, which prime IL-10-producing Tregs (71) (Table 2). Acetate conditions DCs to produce RA in a GPR43-dependent manner, leading to IgA production by B cells (72). Interestingly, unlike acetate, butyrate that induces DC production of RA fails to generate IgA-producing PCs (72). The functional differences between these SCFAs in inducing IgA remain to be elucidated. In addition, commensal bacteria-derived lactate and pyruvate bind to GPR31 and induce dendrite protrusion of CX_3_CR1^+^ LP macrophages, effectively capturing enteric pathogens (73) (Table 2). Of note, macrophage expression of GPR31 and recruitment to the epithelium is induced by CX_3_CR1-CX_3_CL1 interaction between macrophages and iECs (61, 73). Live bacteria also condition DCs. Under homeostatic conditions, commensal bacteria *Enterobacter* and *Alcaligenes* species survive within intracellular compartment of GALT DCs for several days and condition DCs to produce TGF-β, BAFF, IL-6, inducing IgA synthesis (13, 69) (Table 2).

### Conditioning of DCs by IECs

iECs are heterogeneous populations composed of enterocytes, enteroendocrine cells, goblet cells, tuft cells, Paneth cells, and M cells, all of which are derived from intestinal stem cells (ISCs) at the crypt bottom (1). Paneth cells and mesenchymal cells around intestinal crypts express various types of Wnt, an essential factor for the maintenance of ISCs (79). Interestingly, Wnt/β-catenin signaling imprints DCs and macrophages to become tolerogenic, and they produce RA, IL-10, and TGF-β (74). In addition, goblet cell-derived mucus and mucus-coated bacteria also conditions CD103^+^ DCs to produce IL-10, TGF-β, and RA through a galectin-3-dectin-1-FcγRIIB receptor complex that promotes β-catenin signaling, leading to Treg induction (75) (Table 2). The ability of iECs to condition DCs is acquired upon their interplay with commensal bacteria. For instance, SFB anchored deeply into iECs trigger iEC production of serum amyloid A (SAA), which conditions LP DCs to produce IL-6 and IL-23 (76) (Table 2). Commensal bacteria-derived butyrate stimulates iECs to produce RA, thereby inducing tolerogenic DCs (80).

pDCs are a major producer of type 1 IFNs in viral and bacterial infections (57). Interestingly, PP pDCs isolated from naïve mice do not produce type 1 IFNs upon the stimulation with TLR9 ligand (mucosal-type pDCs), due to their local conditioning by TGF-β, IL-10, and prostaglandin E2 produced by iECs and SCs (56). In addition, continuous type 1 IFN signaling during pDC development generates mucosal-type pDCs (81). In this context, we found that the GALT SCs produce constitutively low amounts of type 1 IFNs through continuous stimulation by commensal bacteria, which condition pDCs to express membrane-bound B cell-activating factor belonging to the tumor necrosis factor family (BAFF) and a proliferation-inducing ligand (APRIL), leading to T cell-independent IgA CSR (33). These findings indicate that commensal bacteria condition DCs indirectly through iECs and SCs (Table 2).

## IgA Production in the Intestine

### Mechanism of IgA Synthesis in the Intestine

Murine B cells are divided into conventional B2 cells and primitive B1 cells by different expression of surface molecules CD5, CD11b, and CD23, and their origin, distribution, and antigen-specificity. CD5^−^CD11b^−^CD23^+^ B2 cells (hereafter, B cells) are originated in the BM, and distributed into the secondary lymphoid organs including the GALT, and undergo somatic hyper mutation (SHM) and class switch recombination (CSR) to produce high-affinity antibody with biological effector functions (3, 4). The Ig gene rearrangement is mediated by activation-induced cytidine deaminase (AID), which is induced by activation signals via B cell receptor (BCR), TLRs, CD40, and CD40-related molecules, i.e., BAFF and APRIL (3, 4, 82). In contrast, fetal liver-derived B1 cells are subdivided into CD5^+^CD11b^+^CD23^−^ B1a and CD5^−^CD11b^+^CD23^−^ B1b subsets, both of which are distributed to the peritoneal cavity, thoracic cavity, and intestinal LP, where they undergo limited SHM and CSR to produce low-affinity antibodies (83).

**Figure 1:**
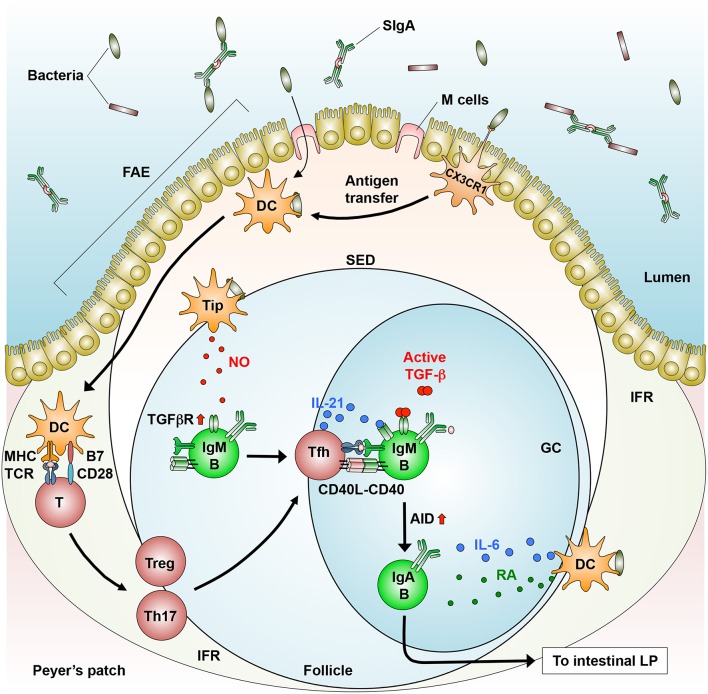
T cell-dependent generation of IgA^+^ B cells in the GALT. In the PPs, dendritic cells (DCs) that engulf directly or indirectly (via antigen transfer by CX_3_CR1^+^ cells) luminal bacteria produce IL-6 and move from the SED to the IFR, where they prime CD4^+^ T cells to generate follicular helper T (Tfh) cells, which are derived from Tregs and Th17 cells. Tfh cells move into the follicle, where they interact with IgM^+^ B cells in a cognate manner (MHC-TCR and CD40-CD40L). In addition, Tip-DCs induce the expression of TGF-β receptor (TGFβR) through their production of nitric oxide (NO). Subsequently, B cells differentiate into IgA^+^ B cells through AID expression in response to TGF-β, IL-21 (produced by Tfh cells), and RA (produced by DCs). IgA^+^ B cells migrate into the intestinal lamina propria (LP), where they differentiate into IgA-producing plasma cells.

IgM^+^ naive B cells acquire surface IgA by undergoing CSR from Cμ (encoding IgM) to Cα (encoding IgA) in the constant region of the Ig heavy chain after they arrive at the PPs. To this end, naive B cells interact with antigen-primed CD4^+^ follicular helper T (Tfh) cells in the light zone of PPs and then move toward the dark zone of PPs to complete both CSR and SHM (3, 4) (Figure 1). In this process, IgA CSR can be accomplished in the absence of T cells as described later. During the SHM and CSR, B cells that express C-X-C motif chemokine receptor 5 (CXCR5) are retained within the dark zone of PPs through the interaction with its ligand CXCL13, which is produced by follicular DCs and SCs (84). B cells that have completed IgA CSR exit the PPs in a S1P- and CXCR4-dependent manner and migrate into the MLNs in a CCR7-dependent manner (85, 86), where DC- and SC-derived RA imprints gut-homing specificity onto the IgA^+^ B cells. The IgA^+^ B cells including plasmablasts home into the intestinal LP, where they differentiate into IgA-producing plasma cells (3, 4, 14, 17). Dimeric or polymeric IgA binds to polymeric immunoglobulin receptor (pIgR), which is a precursor of secretory components on the basolateral surface of iECs, and the IgA-pIgR complex is transported via transcytosis into their apical surface, where the portion of pIgR in the complex is proteolytically cleaved to release into the intestinal lumen as a secretory IgA (3).

### T Cell-Dependent IgA Synthesis

T cell-dependent (TD) IgA CSR takes place largely in follicular B2 cells that reside in the GCs of PPs, where antigen-specific high-affinity antibodies are induced in response to enteric microbes including pathogens (3, 4) and atypical commensal bacteria such as SFB (5, 8). These microbes enter into the SED through M cell transcytosis and direct sampling by macrophages (39, 58, 59). Some macrophages may transfer bacterial antigens to DCs that migrate into the IFR, where they prime T cells (58, 65) (Figure 1). In the GCs, IgM^+^ naïve B cells differentiate into IgA^+^ B cells upon their stimulation by CD40 ligand on activated T cells and by TGF-β1 expressed by multiple cell-types, including iECs, DCs, SCs, and T cells (3, 4, 87) (Figure 1). Furthermore, these processes are promoted by additional factors, as described below.

The GALT contains various types of CD4^+^ T cells. Among them, Tfh cells that express CXCR5, programmed cell death 1 (PD-1), and IL-21 play a pivotal role in inducing TD IgA CSR (88, 89). In the PPs, Tfh cells migrate and localize to the edge of B cell follicles, where they stimulate activated B cells with IL-21 to promote IgA CSR. This migration process is regulated by the interaction of CXCR5 on Tfh cells with its ligand CXCL13, which is secreted from follicular DCs (87). PD-1 plays an integral role in inducing an appropriate IgA repertoire in the PPs through the maintenance of Tfh cell number, leading to homeostatic interaction with the commensal bacteria (90) (Figure 1). However, the mechanisms by which Tfh cells regulate IgA repertoire remain unclear.

Tfh cells are derived from Tregs and Th17 cells in the PPs (91, 92). In an adoptive transfer experiment using T cell-deficient mice, transferred Tregs induce the formation of GCs in the PPs, where they differentiate into IL-21-producing Tfh cells through a down-regulation of Foxp3 and reciprocal up-regulation of Bcl6, a transcription factor critical for CXCR5 induction (91). In a separate study, Th17 cells that were adoptively transferred into T cell-deficient mice were converted into Tfh cells in the PPs (92) (Figure 1). These findings led us to ask which factors convert Th17 cells into Tfh cells in the PPs. In this regard, the conversion of Th17 cells into Tfh cells is induced by commensal bacteria-derived TLR2 ligands that activate T cell-intrinsic MyD88 signaling, leading to the induction of antigen-specific high-affinity IgA (93). The relative contribution of Tregs and Th17 and the role of DCs in the conversion into Tfh cells remain unknown.

### T Cell-Independent IgA Synthesis

**Figure 2:**
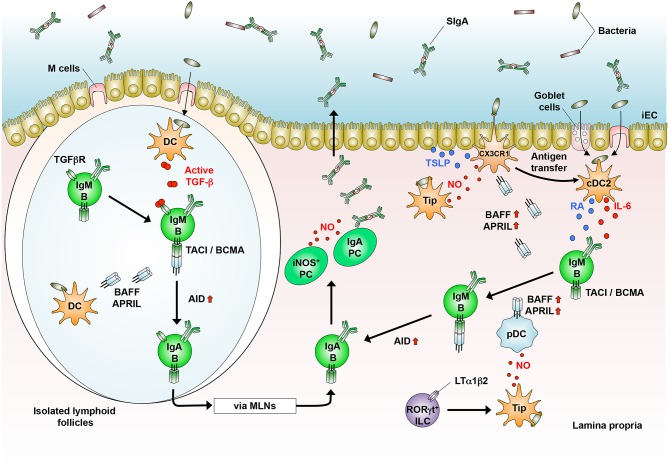
T cell-independent generation of IgA^+^ B cells in the GALT. In the isolated lymphoid follicles, dendritic cells (DCs) that sample luminal bacteria produce TGF-β. DCs also produce BAFF and APRIL to generate IgA^+^ B cells that home into the lamina propria (LP) and differentiate into IgA-producing plasma cells (PCs). In the LP, CD103^+^ cDC2 that sample luminal bacteria produce IL-6 and retinoic acid (RA). CX_3_CR1^+^ cells produce BAFF and APRIL in response to nitric oxide (NO, produced by Tip-DCs and PCs) and TSLP [produced by intestinal epithelial cells (iECs)]. RORγt^+^ innate lymphoid cells (ILCs) induce Tip-DCs in an LTα1β2-dependent manner, which produce BAFF and APRIL by pDCs. In the presence of BAFF/APRIL, RA, and IL-6, IgM^+^ B cells generate IgA^+^ B cells, which differentiate into IgA-producing PCs.

Quantities of IgA in the sera and intestinal secretions are somewhat reduced in mice lacking T cells or CD40 that are critical for T cell help and GC formation (33, 42, 94–96), indicating that IgA CSR can also be induced in T cell- and GC-independent manners (Figure 2). In addition, SHM is not completely accomplished in the Ig variable regions of these mice, leading to induction of low-affinity IgA (95, 96).

T cell-independent (TI) IgA CSR occurs in B1 cells, especially B1b cells, and extrafollicular B2 cells of the GALT, where homeostatic IgA is constitutively produced by the stimulation with commensal bacteria (5, 33, 94, 96–102). In this context, CD103^+^ cDC2 capture these bacteria that are transported into the LP through transcytosis by M cells and goblet cells and direct sampling by macrophages (60–64), leading to TI IgA synthesis (Figure 2). In general, TI IgA production is predominantly mediated by B1b cells (5, 102). In this context, approximately 40% of the total IgA-producing plasma cells in the LP are of peritoneal B1 cell origin (83, 94, 101). Upon stimulation with TLR2 ligands, the B1 cells exit the peritoneal cavity by downregulating CD9 and integrins α1, α6, and β1, and some of them migrate into the LP (103). Similar to peritoneal B1 cells, conventional B2 cells also produce IgA independently of T cells upon the stimulation with BAFF and APRIL, which are both structurally similar to CD40 ligand (33, 42, 99, 100, 104), or their stimulation with TI antigens, TLR ligands, and possibly TGF-β (105) (Figure 2).

The homeostatic IgA contributes to the maintenance of commensal bacterial homeostasis. *Tcrb*^−/−^*Tcrd*^−/−^ mice, which lack T cells, have IgA antibodies that are reactive to commensal bacteria, suggesting that the production of the IgA is mediated by the TI pathway (5, 94). In addition, the homeostatic IgA can eliminate some pathogens such as rotavirus and *S. typhimurium* until pathogen-specific high-affinity IgA is secreted into the intestinal lumen (106, 107), implying that the TI pathway temporarily substitutes for the TD pathway during the early phase of infection.

## Role of Dendritic Cells in Intestinal IgA Production

In early studies, the induction of IgA production by DCs was demonstrated by using co-culture systems including B cells, T cells, and DCs. Human DCs enhance the IgA production by CD40L-stimulated B cells (108, 109). In mice, CD11b^+^ cDC2 isolated from the PPs can preferentially induce IgA production in the presence of T cells and antigens, and this process requires IL-6R signaling (110) (Figure 1). The PP DCs carrying live commensal bacteria induce IgA production by B cells in either the presence or the absence of T cells (13), confirming that intestinal DCs are capable of inducing IgA CSR in both TD and TI manners. DC-associated molecules that induce IgA synthesis are as described below.

### TGF-β

Although TGF-β is constitutively expressed in multiple cell types, its production is tightly regulated (111). In brief, TGF-β is first synthesized as an inactive latent complex with latency-associated peptide, and this complex cannot bind the TGF-β receptor. The latent complex is then cleaved by matrix metalloproteinases (MMPs) and/or integrins to release active TGF-β. DCs, macrophages, and follicular DCs in the GALT produce TGF-β through their expression of integrin αvβ8 and MMP2/9/13, all of which are up-regulated by the stimulation with TLR ligands and RA (100, 112, 113). As physiological TLR ligands and RA are enriched in the GALT, these machineries may explain the establishment of TGF-β-abundant microenvironment in the GALT.

### Retinoic Acid

In the intestine, RA is mainly derived from dietary and bile retinol (vitamin A). In brief, retinol is oxidized to retinal by alcohol dehydrogenase and further to RA by aldehyde dehydrogenase (ALDH). DCs in the PPs and MLNs express ALDH1 (encoded by *Aldh1a1*) and ALDH2 (encoded by *Aldh1a2*), respectively, and induce IgA synthesis in an RA-dependent manner in the presence of IL-5 and IL-6 (15, 114). Similarly, CD103^+^ LP cDC2 expressing TLR5 induce IgA CSR in peritoneal B cells through the production of RA and IL-6 in the presence of flagellin (28) (Figure 2). These findings indicate that intestinal DC-driven TI IgA production is largely dependent on RA. However, RA alone is insufficient to induce IgA CSR in naïve B cells (115).

### BAFF and APRIL

BAFF and APRIL are produced as either soluble or membrane-bound form by DCs and their related cells upon their stimulation with TLR ligands, type I IFNs, IL-10, and TSLP (104, 105, 116). Under steady-state conditions, intestinal DCs produce large amount of BAFF or APRIL that directly induces IgA CSR *in vitro* and promote the survival of post-switched IgA^+^ B cells and IgA-producing plasma cells in the GALT (42, 52, 105) (Figure 2). In addition to DCs, some intestinal CX_3_CR1^+^ macrophages also induce IgA production in a BAFF/APRIL-dependent but TLR- and RA-independent manner (117) (Figure 2). In humans, IgA2 CSR, which is predominant in the colon, is dependent on APRIL that is derived from iECs rather than from DCs (99), indicating that the major source of APRIL differs between humans and mice. Transmembrane activator and calcium-modulating cyclophilin-ligand interactor (TACI) and B cell maturation antigen (BCMA) are common receptors for BAFF/APRIL (116). In this context, TACI signaling does not involve SMADs (SMAD2/3/4) and RUNX3, which bind to the TGF-β responsive element in Iα promoters that is essential for IgA synthesis (3, 118), suggesting that BAFF and APRIL probably activate Iα promoters in a TGF-β receptor-independent manner. However, DC-driven TI IgA production is partially inhibited by the neutralization of TGF-β (52, 105), suggesting that TGF-β is additionally involved in and optimizes BAFF/APRIL-induced IgA CSR. The serum IgA level is selectively decreased in mice lacking APRIL or TACI (119, 120), and in patients carrying TACI mutations (121), confirming that these cytokines are important for TI IgA production. Consistently, IgA production from B cells of mice lacking TACI and BCMA is impaired when co-cultured with PP DCs from wild-type mice or stimulated with soluble BAFF and APRIL (52, 119). In contrast, there is a controversial report that the serum IgA level is not affected in *April*^−/−^ mice (122), implying that differences in the components of commensal bacteria among individual strains of *April*^−/−^ mice and between different animal housing facilities may affect the IgA level, although the precise reason remains unclear.

## Involvement of Nitric Oxide in IgA Synthesis

### Nitric Oxide

Nitric oxide (NO) is a free radical molecule synthesized from L-arginine by three different isoforms of NO synthase (NOS): neuronal NOS (encoded by *Nos1*), inducible NOS (iNOS, encoded by *Nos2*), and endothelial NOS (encoded by *Nos3*) (123). Since gaseous NO is a small diffusible molecule, it readily penetrates the microenvironment, where it functions as a neurotransmitter, an immune modulator, and a vasodilator. Furthermore, since NO can passively penetrate cellular membranes, its main targets are intracellular proteins. NO exerts its biological effects through at least two pathways: heme iron- and S-nitrosylation (123). In the former pathway, NO activates soluble guanylate cyclase (sGC) through iron nitrosylation of the heme group, and the sGC catalyzes the conversion of guanosine monophosphate (GMP) into second messenger cyclic GMP, which activates protein kinase G. In the latter pathway, NO controls the activity of various intracellular signaling molecules, including enzymes and transcription factors, through S-nitrosylation of the cysteine thiol group (SNO). In general, a low concentration of NO activates cGC and transcription factors, such as NF-κB, whereas a high NO concentration causes the SNO of signaling molecules (123).

### Nitric Oxide Dependency of IgA Synthesis

iNOS is expressed in DCs and macrophages and mediates large amounts of NO production upon its stimulation with bacterial products and inflammatory cytokines, leading to the killing of bacteria. Upon infection with *Listeria monocytogenes*, Ly6C^+^ inflammatory monocytes differentiate into TNF/iNOS-producing cells (previously defined as Tip-DCs) in CCR2- and MyD88-dependent manners (36, 124, 125). In the intestine, the continuous stimulation by commensal bacteria may induce “homeostatic inflammation,” which is essential for IgA production and possibly iNOS expression. Supporting this notion, commensal *B. fragilis*-derived PSA induces iNOS expression and NO production in monocyte-derived DCs (68). In this context, we showed that *Nos2*^−/−^ mice and wild-type mice that treated with iNOS inhibitors have a reduced frequency of IgA^+^ B cells in the GALT and lower levels of serum and fecal IgA (42). Notably, iNOS-expressing CD11c^+^ cells, which may correspond to the *Listeria*-induced Tip-DCs, are preferentially induced in the GALT of wild-type mice, in a manner involving the MyD88-dependent recognition of commensal bacteria. Interestingly, such “naturally occurring” Tip-DCs in the GALT are of inflammatory monocyte origin, because they are absent in the GALT of *Ccr2*^−/−^ mice (126) (Table 2). In line with these findings, mice lacking *Mcp-1*, a primary ligand for CCR2, show impaired IgA production in the lung upon influenza infections (127). Importantly, the adoptive transfer of Tip-DCs into *Nos2*^−/−^ mice restores IgA levels in the sera and feces. Tip-DCs have the potential to induce IgA synthesis mediated by both TD and TI pathways (42). In the TD pathway, Tip-DC-derived NO induces type II TGF-β receptor on B cells (Figure 1), whereas NO induces the DC expression of BAFF and APRIL in the TI pathway (Figure 2). Interestingly, in the intestine, NO induces DC expression of CCR7, which is essential for their migration into the MLNs (128), and TNF-α is required for the expression of MMPs, which mediate TGF-β activation (100) (Figure 2). Some Tip-DCs express ALDH1 and ALDH2 and an RA response element is located in the promoter region of the *Nos2* gene (129). Indeed, RA-treated DCs, which have tolerogenic properties, induce the expression of iNOS (130), suggesting that microenvironmental conditioning factors, including bacterial and dietary components induce iNOS expression in the intestinal DCs. Furthermore, RORγt^+^ innate lymphoid cells (ILCs) can induce iNOS expression in intestinal DCs through their membrane-bound lymphotoxin α1β2 expression, leading to TI IgA production (131) (Figure 2). Given these observations, it is now clear that iNOS are expressed in some CD11b^+^ DCs and their related cells under the influence of intestinal microenvironments (41, 43, 52, 132), leading in part to the establishment of prominent IgA-producing sites. In addition to intestinal DCs and their related cells, some plasma cells also express iNOS and regulate the composition of the microbiota through an intrinsic NO-dependent IgA production (133). Collectively, “homeostatic” iNOS-derived NO produced by multiple cell types appears to contribute to the maintenance of intestinal homeostasis.

## Extrafollicular DC-B Cell Interaction in IgA Synthesis

**Figure 3:**
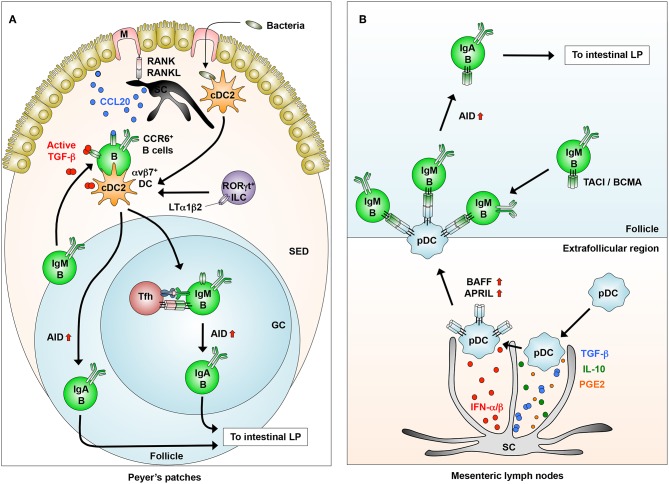
Novel DC-B cell interaction in inducing IgA synthesis. **(A)** In the Peyer's patches, stromal cells (SCs) interact with M cells to produce CCL20, which induces the migration of IgM^+^CCR6^+^ B cells from the follicles into the SED through the CCR6-CCL20 interaction. B cells that migrated into the SED interact with dendritic cells (DCs) that express integrin αvβ8, which is induced by LTα1β2 from RORγt^+^ innate lymphoid cells (ILCs). B cells activated in the SED migrate into the follicles to generate IgA^+^ B cells through the induction of AID. **(B)** In the extrafollicular region of the mesenteric lymph nodes, SCs condition pDCs to express membrane-bound BAFF/APRIL through their production of IFN-α/β, albeit at low levels, and possibly TGF-β, IL-10, and PGE2. Conditioned pDCs make close contact with IgM^+^ B cells through BAFF/APRIL-TACI/BCMA interaction to induce AID expression for IgA class-switching.

Recently, the SED of the PPs has attracted attention as a new site for IgA CSR. In the SED, mesenchymal SCs located close to the FAE express membrane-bound receptor activator of nuclear factor-κ B ligand (RANKL), which is essential for epithelial CCL20 production and M cell differentiation through binding to its receptor RANK (134) (Figure 3A). Under steady-state conditions, pre-GC IgD^+^ B cells that express CCR6 are recruited in a CCL20-dependent manner into the SED, where they are in close contact with cDC2 that have engulfed luminal antigens transported through M cells, thereby initiating IgA CSR (134). In parallel, the close interaction of B cells with the cDC2 expressing αvβ8 that activate TGF-β promotes IgA CSR in the SED (135) (Figure 3A). In this context, Tip-DCs and pDCs are predominant in the SED of the PPs (56, 126), implying that they interact with B cells in a different fashion. In the SED, group 3 ILCs condition αvβ8^+^ cDC2 (Figure 3A) and possibly Tip-DCs to induce IgA synthesis through their expression of lymphotoxin α1β2 (131, 135). B cells activated in the SED move back to the GCs, where some of them appear to complete their differentiation into IgA^+^ B cells through TD signaling (6, 135) (Figure 3A).

We previously reported that pDCs in the GALT have the potential to induce TI IgA CSR in a membrane-bound BAFF/APRIL-dependent manner (33) (Figures 2, 3B). Interestingly, the pDCs expression of BAFF/APRIL is induced by type 1 IFNs produced, albeit at low levels, from intestinal SCs and by NO from naturally occurring Tip-DCs. The SC expression of type I IFNs is largely dependent on the stimulation by commensal bacteria (33) (Figure 3B).

## Mucosal Vaccines

As the mucosa is the major entry site for most pathogens, IgA secreted in the mucosal lumen has an important role in preventing their penetration across epithelial barriers. Thus, the efficient induction of antigen-specific IgA in the lumen has led to successful mucosal vaccines such as oral and nasal vaccines that are more effective than conventional intramuscular and subcutaneous vaccines. However, mucosal vaccines for human use are now available for only a few pathogens, due to the fact that their safety has not been demonstrated and to a lack of effective adjuvants and delivery systems (136). Although the mucosal vaccines that are currently licensed for human clinical trials consist of either live attenuated or inactivated pathogens, some safety issues remain unresolved. In contrast, subunit vaccines composed of molecules from pathogens are typically safer, but offer less powerful immunogenicity due to their susceptibility to digestion (136). In this regard, the establishment of delivery systems to DCs within the GALT, i.e., PPs, is important. Given that M cells transport and deliver luminal antigens to DCs in the PPs, M cells and their surface molecules i.e., GP2, are possible targets for effective vaccine delivery (137). Indeed, the oral administration of anti-GP2-conjugated salmonella antigen increases the host's resistance against *S. typhimurium* infection in mice (138).

The generation of long-living IgA-producing plasma cells is also required for successful mucosal vaccines. In mice, the maximum lifespan of intestinal IgA-producing plasma cells is around 7–8 weeks (average half-life of 4–5 days) even under steady-state conditions (139). The maintenance of long-living plasma cells in the intestine appears to be mediated by BAFF, APRIL, and IL-6. In addition, plasma cell-intrinsic iNOS-derived NO increases their survival and longevity in non-mucosal and mucosal tissues (133, 140). Given that Tip-DCs produce gaseous NO (42, 124), they may complementarily prolong and further enhance the survival of iNOS^−^ and iNOS^+^ plasma cells, respectively. In addition, commensal bacteria-derived lactate and pyruvate condition intestinal macrophages to induce dendrite protrusion, by which macrophages sample enteric pathogens in the lumen (73), suggesting that these would be good candidates for mucosal adjuvants that target DCs and macrophages.

In contrast to inbred mice, immune responses in humans vary between individuals and there are evaluation limits of clinical trials, suggesting the need for appropriate animal models for the evaluation of mucosal vaccines. In this regard, the use of non-human primates that are physiologically and immunologically similar to humans is reasonable. Recently, rhesus macaque models have been used to show that mucosal immunization with a vector expressing simian immunodeficiency virus (SIV) protein increases the numbers of pDCs and myeloid DCs in both the rectal mucosal tissues and the blood, thereby inducing effective TD mucosal immune responses (141, 142). Importantly, these rectal DCs produce large amounts of BAFF, IL-6, and TNF-α to induce SIV-specific IgA production (141). In addition, given the limited availability of non-human primates, the development of humanized mice having human immune systems and microbiota might be useful in overcoming some of these problems.

As described, homeostatic IgA shapes the composition and diversity of commensal bacteria, leading to the maintenance of host health (6–9). Indeed, “mild” dysbiosis is induced in IgA-deficient mice and patients with IgA-deficiency (7–9, 143). In both humans and mice, antibiotic-induced dysbiosis causes various diseases such as colitis, allergy, autoimmunity, obesity, autism, and infection (144). Importantly, intact composition of commensal bacteria prevents colonization by pathogens in the gut (145, 146) and homeostatic IgA eliminates some pathogens (106, 107), implying that homeostatic IgA contributes to host defense. In this context, the TI pathway that is critical for homeostatic IgA production is similar between humans and mice, except for the source of colonic APRIL (3, 4, 99), suggesting that a thorough understanding of the TI pathway should contribute to effective production of homeostatic IgA, which regulates the healthy balance of gut microbes. Accordingly, not only the induction of pathogen-specific IgA mediated by the TD pathway but also the maintenance of homeostatic IgA production by the TI pathway may present new pathways for the development of mucosal vaccines.

## Concluding Remarks

Accumulating evidences have been revealing that microenvironmental factors, in particular commensal bacteria, condition DCs to acquire their mucosal phenotype with tolerogenic and IgA-inducing properties in the GALT. However, little is known where and how individual or a group of commensal bacteria condition DCs to induce intestinal IgA synthesis. In this regard, we need to understand about the characteristics of commensal bacteria that are preferentially engulfed by intestinal DCs or that stimulate iECs to produce conditioning factors for DCs.

In addition, we are just beginning to understand the role of the close interaction between DCs and B cells in inducing IgA synthesis in the extrafollicular foci such as the SED of the PPs. Such collaborative actions between the conditioned DCs and B cells before their interaction with T cells appear to be required for the development of mucosal vaccines that will induce effective immune responses, including antigen-specific and long-lasting IgA production. In this context, commensal bacteria-derived metabolites that condition DCs to induce long-living IgA^+^ plasma cells or to promote antigen sampling may be promising adjuvants in the development of safe and effective mucosal vaccines. Therefore, understanding the mechanisms of these processes and their regulation will facilitate the development of mucosal vaccines.

## Author Contributions

All authors listed have made a substantial, direct and intellectual contribution to the work, and approved it for publication.

### Conflict of Interest Statement

The authors declare that the research was conducted in the absence of any commercial or financial relationships that could be construed as a potential conflict of interest.
